# Literature Lab: a method of automated literature interrogation to infer biology from microarray analysis

**DOI:** 10.1186/1471-2164-8-461

**Published:** 2007-12-18

**Authors:** Phillip G Febbo, Mike G Mulligan, David A Slonina, Kimberly Stegmeir, Dolores Di Vizio, Paul R Martinez, Massimo Loda, Stephen C Taylor

**Affiliations:** 1 Institute for Genome Science and Policy, Duke University, Durham, North Carolina, USA; 2 Department of Medicine, Duke University School of Medicine, Durham, North Carolina, USA; 3 Department of Molecular Genetics and Microbiology, Duke University School of Medicine, Durham, North Carolina, USA; 4 Acumenta Corporation, Boston, Massachusetts, USA; 5 Department of Pediatric Oncology, Dana-Farber Cancer Institute, Harvard Medical School, Boston, Massachusetts, USA; 6 Department of Pathology, Dana-Farber Cancer Institute, Harvard Medical School, Boston, Massachusetts, USA

## Abstract

**Background:**

The biomedical literature is a rich source of associative information but too vast for complete manual review. We have developed an automated method of literature interrogation called "Literature Lab" that identifies and ranks associations existing in the literature between gene sets, such as those derived from microarray experiments, and curated sets of key terms (i.e. pathway names, medical subject heading (MeSH) terms, etc).

**Results:**

Literature Lab was developed using differentially expressed gene sets from three previously published cancer experiments and tested on a fourth, novel gene set. When applied to the genesets from the published data including an *in vitro *experiment, an *in vivo *mouse experiment, and an experiment with human tumor samples, Literature Lab correctly identified known biological processes occurring within each experiment. When applied to a novel set of genes differentially expressed between locally invasive and metastatic prostate cancer, Literature Lab identified a strong association between the pathway term "FOSB" and genes with increased expression in metastatic prostate cancer. Immunohistochemistry subsequently confirmed increased nuclear FOSB staining in metastatic compared to locally invasive prostate cancers.

**Conclusion:**

This work demonstrates that Literature Lab can discover key biological processes by identifying meritorious associations between experimentally derived gene sets and key terms within the biomedical literature.

## Background

The accelerating expansion of biomedical research outpaces most individual attempts at comprehensive review even in relatively narrow fields. Just as the vast sequence data available for the human [1,2] and additional organisms [3-5] require sophisticated genomic browsing tools [6-8], computational methods are required to thoroughly explore the corpus of biomedical literature. Many computational methods for interrogating the scientific literature have been developed [9]. These programs can be broadly defined as methods for information retrieval and those for information extraction [10].

Existing methods can identify significant association between individual genes and terms from the medical subject heading (MeSH) index and Gene ontology (GO) databases [11], manually curated biological lists [12], or disease-specific lists [13]. These prior methods demonstrate that genes with disease-specific differential expression can be strongly correlated with key terms within the medical literature [13,14]. In addition, gene-gene associations within the literature have been combined with multiple available databases to extend associations beyond the literature alone [15].

While these and similar approaches have underscored the potential of automated literature searching to facilitate discovery, few have provided both methods for assessing the statistical strength of identified associations and supported their methods with experimental validation. Here, we describe and apply "Literature Lab", a method of automated data retrieval confined to publicly available citations and abstracts. Literature Lab statistically assesses identified associations within the corpus of medical literature between sets of experimentally derived genes and key terms derived from curated or MeSH lists. We demonstrate that our methodology can identify previously reported relationships and can result in discovery.

### Molecular Methods

#### Gene Expression Sets

Literature lab was applied to three gene sets derived from previously published microarray data and one gene set from as yet, unpublished dataset to determine if literature mining identified important metabolic, physiologic, or pathway activity. These gene sets include, 1) the top 100 genes with increased expression during *in vitro *exposure of human leukemia cells (HL60, AML cell line) to ATRA ("UCT"), 2) the genes differentially regulated with a transgenic model of prostate neoplasia (MPAKT) following exposure to RAD001, 3) the 70 genes used to predict outcome in localized node-negative breast cancer, and 4) genes differentially expressed between malignant epithelial cells in local v. metastatic prostate cancers. The genes lists are included in additional files and a full description of the development of each gene set is in the supplemental methods (see Additional File 1).

#### Western Blots

Protein from fresh ventral prostates were extracted in RIPA buffer [10 mM sodium phosphate (pH7.2) 150 mM NaCl, 1% Nonidet P-40, 0.1% SDS, 1 mM NaOVa, 1 mM DTT, 5 mM NaF, 0.1% sodium deoxycholate, 10 μg/ml leupeptin, 10 μg/ml aprotinin, and 1 mM PMSF] separated by gel electrophoresis and transferred to nitrocellulose membrane (0.45 μM) as described[16,17]. Membranes were blotted with anti-Hif-1α (kindly provided by J. Pouyssegur) and anti-tubulin (B-5-1-2) (Sigma) (1:1000). Blots were scanned and intensities were measured.

#### Immunohistochemistry

A prostate tissue microarray containing samples of benign prostate epithelium (n = 14), locally invasive prostate adenocarcinoma (n = 20), and metastatic prostate adenocarcinoma (n = 22) were stained for FOSB. The FOSB staining was performed as previously described [18,19]. Briefly, 5 micrometer sections were cut from the TMA block, deparaffinized, rehydrated and subjected to microwaving in 10-mM Citrate buffer (pH 6.00) in a 750W oven, for 15 minutes. The polyclonal anti-FOSB primary antibody (Santa Cruz Biotechnologies, Inc.), was incubated (1:50 dilution) at room temperature in an automated stainer (Optimax Plus 2. 0; Biogenex, San Ramon, CA). Antigen-antibody reaction was revealed with standardized development times, using the Streptavidin method with 3, 3 diaminobenzidine as substrate. Meyer Hematoxylin was used as nuclear counterstaining. FOSB nuclear positivity was scored on a total of 50 nuclei per sample. The statistical difference in FOSB staining between the unpaired populations was assessed using a two-tailed Mann Whitney test (GraphPad Prism4 Software).

#### Application of Established Literature Mining Tools

MeSHer data for the gene lists was obtained from the website [20] by supplying the Affymetrix U133Plus2 Ids corresponding to the list of genes using a p-value threshold of 1.0 and no p-value correction.

GOMiner data for the gene lists was obtained by following the instructions for downloading (build 148) and installing the GOMiner application and SQL database from [21]. A list of gene symbols for genes in the Affymetrix U133Plus2 probe set list was used for the 'Total' file and lists of symbols for the experimental genes used for the 'Changed' file. Other GOMiner settings used were the default values defined in the GOMiner application.

## Implementation

### Overview

To highlight and prioritize cellular physiology, metabolism, or pathways differentially active within a microarray experiment, Literature Lab uses an experimentally derived gene list, pre-defined sets of non-ambiguous key terms ("domains"), inclusive gene nomenclature, comprehensive literature interrogation, and a comparison of the experimental gene set results with randomly generated gene sets. For each experimentally derived gene set, Literature Lab performs an automated literature search to determine the number of abstracts listing any of the identified genes and each term within the term list (or domain). Specific domains include those for cell metabolism (MeSH headings), cell physiology (MeSH headings), and cellular pathways (manually curated from multiple sources) (Additional File 2). Terms are ranked with a measure of association between genes and terms known as the product of frequency (PF) (see Additional File 1). The strength of the relationship between an experimental gene set and a term is determined by comparing the log (base 10) of the sum of the PF values for the experimental gene set (called the LPF) with a distribution of the same values for 1000 random gene sets of the same size as the experimental gene set. A score representing the number of standard deviations above or below the mean value for the random gene sets is assigned to each term and terms are ranked in decreasing order of this score. To highlight terms with particularly strong association with the gene list, we developed heuristic rules for labeling the relationships as "Strong" or "Moderate" (see Additional File 1 for Details) (the software used for these analyses is free for non-commercial use and available, see Availability and Results section).

### Overall Architecture of the Software Implementation

The software is implemented using Sun's Java programming language [22] using the Eclipse software development environment [23]. The software consists of three major components:

• A series of programs which query PubMed for each term and each gene in the Gene Thesaurus using NCBI's Entrez Programming Utilities [24] and Sun's JAXB XML Binding classes [25]. The result for each term or gene is a file containing a list of the PubMed Ids for the term or gene. In addition, summary files containing counts of the number of abstracts for each gene and for each term are prepared.

• A series of programs which identify the PubMed Ids in common for each term/gene combination from the lists of ids for each term and each gene. The results of these programs are files containing the PubMed Ids for each term/gene combination and files containing summary counts of the number of abstracts for each term/gene combination.

• A series of programs for preparing the analysis of a specific gene list. These programs compute the described statistics for the gene list and for one thousand random gene sets of the same size as the specific gene list being processed. The final step of this process prepares a Microsoft^® ^Excel spreadsheet containing the results of the analysis. The Apache Software Foundation's POI classes (Java API to Access Microsoft^® ^Format Files [26] are used to format the data in a form which can be viewed in Microsoft^® ^Excel.

### Gene Annotation

Gene annotation began by obtaining gene names, symbols, and "aliases" from Stanford University's SOURCE web site [27]. Subsequent Boolean PubMed searches and manual review for precision (i.e. the ability to accurately identify abstracts related to the gene of interest) were used to develop a set of rules required for more specific automated searching (Additional File 1). In subsequent literature searches, gene terms consisting entirely of numerals, of three characters or less, or that were identified as excessively ambiguous through manual review were excluded. Algorithm-based disambiguation was not used [28]. While the database of gene terms (referred to as the gene thesaurus) is frequently updated, for all experiments herein described, the gene thesaurus updated last on 12/30/2003 was used for all experiments presented (Additional File 3). 514 of the gene terms were specifically excluded based upon the manual review (Additional File 4). All gene term curation was performed prior to testing for associations.

### Topic List Curation

Topic lists are sets of terms, each of which is to be tested for significant associations with the experimentally derived gene set. Topic lists used in the experiments were defined using terms from the MeSH Thesaurus provided by the National Library of Medicine. The list of terms for each topic set consisted of all terms and all descendents (with few exceptions) as listed in MeSH. The topic sets (presented as "name [Mesh ID]") used in these experiments were Cell Metabolism [G06.535] and Cell Physiology [G04.335] (Additional File 2). A few of the descendents of these MeSH terms were ignored because very few abstracts were associated with the term. When using MeSH terms to search PubMed, the search syntax used "MeSH Subheading Explosion" so that the resulting list of abstracts included abstracts coded with descendents (if any) of the MeSH term as well as abstracts coded with the MeSH term itself.

In addition, a topic set entitled "Pathways" was derived using the pathway descriptions from BioCarta [29] with some additional curation and testing (see Additional File 1 for methods and Additional File 2 for list of pathways). All pathway term curation was performed prior to testing for associations.

### Literature Search

A list of the PubMed abstract ID's for each gene and each topic were obtained by searching PubMed using the appropriate terms and a constant date range. The PubMed Electronic Date (EDAT) was used to query a constant subset of the PubMed abstracts between 12/31/93 and 12/31/03. Whole text information was not used due to its incomplete and inconsistent availability. To determine if results changed over time with a fixed chronological time frame and pre-set search parameters, three gene lists ("Ideal", "UCT", and "Van't Veer") and three curated term lists (metabolism, physiology, pathway) were run repetitively every month for 5 months using the fixed dates above in order to assess if ongoing efforts at the NCBI to improve the search engine or annotation of the literature significantly influence our approach to literature mining.

### Term Ranking

Each topic list was analyzed independently. Within each topic set, specific terms were ranked according to how many times the term was associated with any of the genes in the gene list with respect to the total number of times the term is present in PubMed. We applied two different methods to measure the degree of intersection between any term and the gene set. First, we calculated the ratio of the number of abstracts containing any gene from the gene list AND the term divided by all abstracts containing the term (referred to as "expected abstracts"). The second score (called "product of frequency") takes into account the number of abstract mentions against the entire target gene set (see Additional File 1). In order to rank terms, we compared each terms score (by either method) to a distribution of scores between the same term and randomly selected gene sets. In the case of the product of frequency, the log(PF) (LPF) more closely approximated a normal distribution thus justifying the mean and standard deviation statistics used (see Additional File 1).

### Testing against random gene sets

In order to provide a metric by which to interpret the rankings and determine the likelihood of finding a match given no association, we measure each topic score given the experimental set of genes against the distribution of scores from sets of genes chosen at random. Scoring 1,000 such random sets against the topic set, we obtain estimates for the mean and standard deviation of the *F*(*geneset*, *topic*) score for each topic. We tested if there was a significant difference in the statistics generated using or 1000 random sets of genes containing the same number of genes as the experimental gene set.

## Results

We applied Literature Lab to lists of genes generated through microarray experiments to determine if such an approach provides biological insight. We started with 3 lists of genes from previously published microarray experiments, one generated in vitro (HL60 cells treated with ATRA [30], one generated in vivo (MPAKT transgenic mice treated with RAD001 [17]), and one generated from human tumors samples (A 70 gene model of breast recurrence [31,32]) to see if Literature Lab would correctly identify known biological processes and to how altering specific variables within Literature Lab impacts the results. Finally, we tested our method to a set of genes differentially expressed between local and metastatic prostate tumors and used immunohistochemistry to confirm the lead candidate.

### Literature Lab associates "respiratory burst" with ATRA treatment of a leukaemia cell line

**Figure 1:**
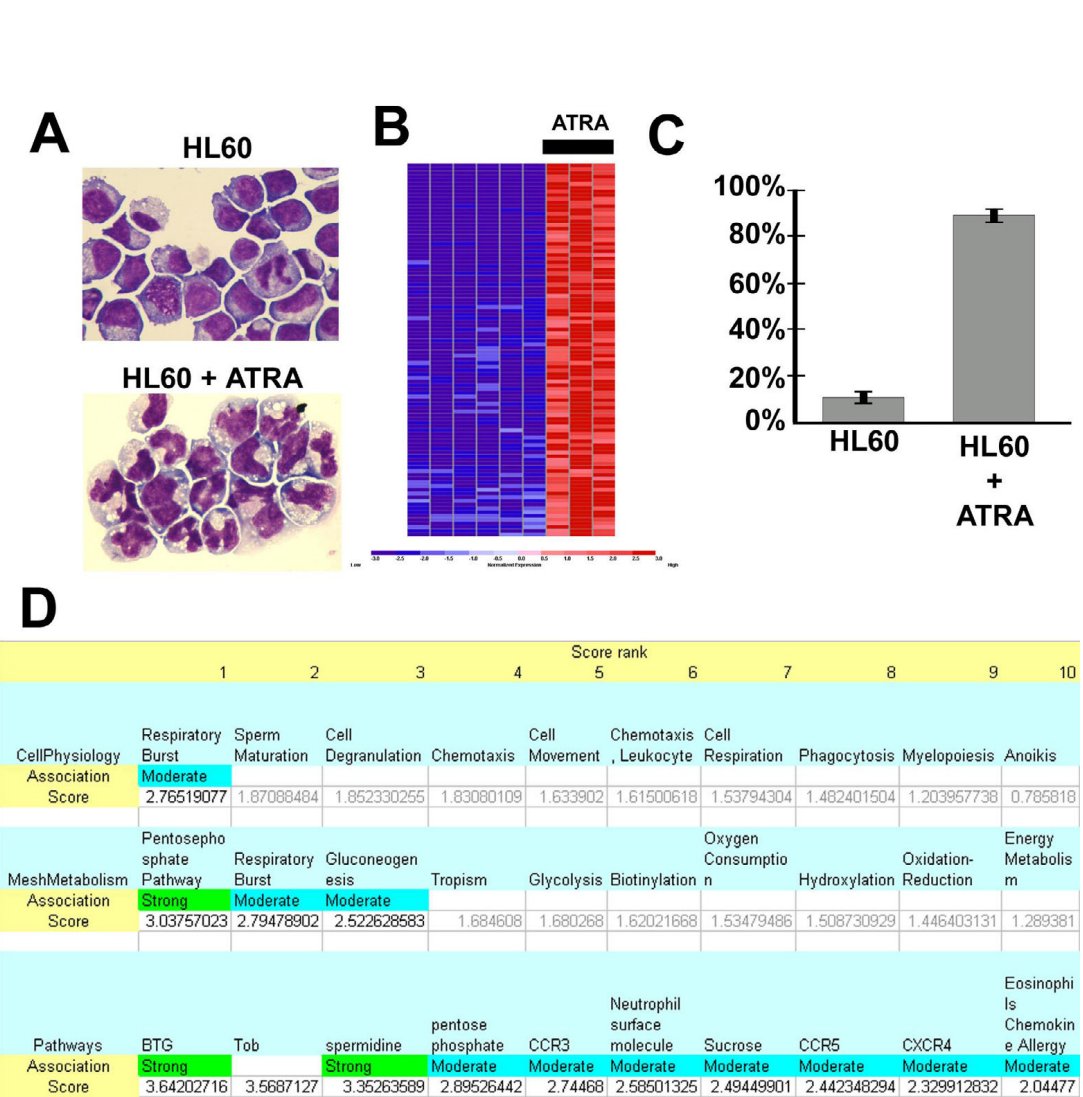
**HL60 Leukemia cells treated with ATRA**. A) May Grunwald Giemsa stain of HL60 cells prior to (upper) and following treatment with ATRA (lower). B) Heat map of the top 100 genes with increased expression in HL60 cells treated with ATRA compared to untreated HL60 cells (Red – high normalized expression, Blue – low normalized expression). C) Percentage of cells positive for nitro blue tetrazolium (NBT) reduction (Mean +/- St dev). D) Literature Lab ranking, association scores, and confidence calls for cell physiology, metabolism, and pathway terms (Association score is the log of the product of frequency (logPF)).

Acute promyeloblastic leukemia (APML) cells differentiate when stimulated with all-trans retinoic acid (ATRA), a life-saving treatment for this disease [33]. The physiological impact of ATRA is detected by an increase in nitro blue tetrazolium (NBT) reduction (Fig. 1D), an assay established to measure the production of oxygen intermediates associated with respiratory bursts in cells [34]. We used microarray data available from a recent publication [30] to identify the top genes with increased expression in HL60 cells (a cell line used to model APML) following ATRA exposure (Fig. 1A and 1B). Genes with increased expression were analyzed with Literature Lab using key term sets for cellular metabolism, physiology, and pathways. Within the top ranked key terms for both metabolism and physiology was "respiratory burst" (Fig. 1C). This observation is confirmed by an increase in nitro blue tetrazolium (NBT) reduction, an established measure of the production of oxygen intermediates associated with respiratory bursts in cells [34].

We tested the impact of gene set size and literature time frame on the successful association between "respiratory burst" and the genes with increased expression following ATRA. While the specific literature time frame had relatively little impact on the association (i.e. first or last 5 years of the 10 year period) (see Additional File 5), the number of differentially expressed genes included in the analysis did impact our results (see Additional File 6) Specifically, the association between "respiratory burst" and the experimentally derived list of differentially expressed genes did not stabilize until greater than 150 genes were included. While there are no obvious rules to guide the upper limit with respect to the number of genes to be used in literature lab, the variability of results observed with shorter gene lists suggests that gene lists numbering less than 25 will not provide robust results and for experiments comparing phenotypes with marked differences, gene lists greater than 150 are encouraged.

### "Hif and Hypoxia" strongly associated with mTOR inhibition

**Figure 2:**
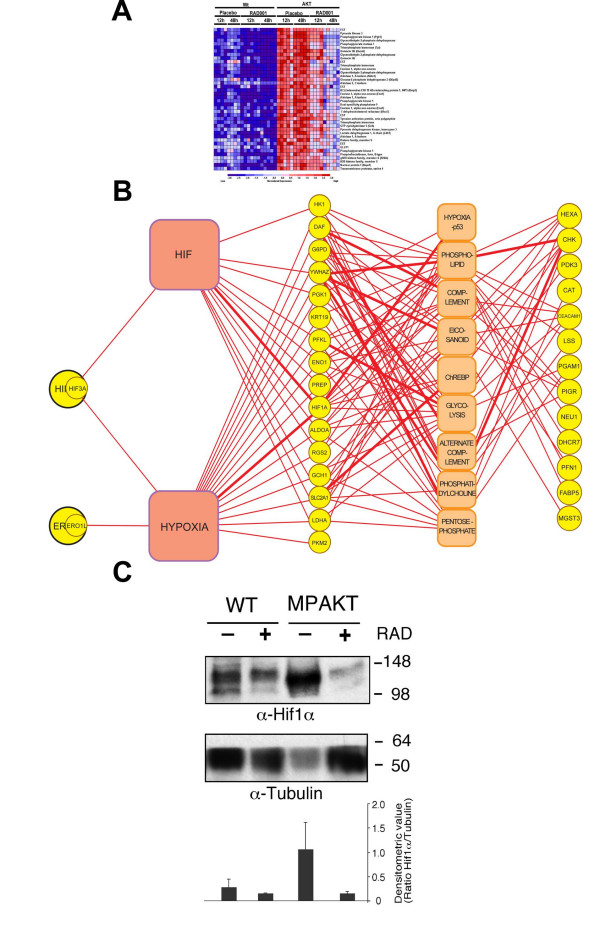
**MPAKT mouse treated with RAD001**. A) Heat map of normalized gene expression for the top 64 genes correlating with AKT expression and RAD001 treatment in the prostates of transgenic MPAKT mice. B) Association of pathway terms (Squares) with the 64 genes (circles) associated with RAD001 treatment of MPAKT mice. Size of square indicates confidence of associations, thickness of connecting line correlates with the number of abstracts linking any pair of term and gene. C) Protein lysates prepared from the VP of individual MPAKT and WT mice either treated with RAD001 (+) or with placebo (-) for 48 hours were immunoblotted with anti-Hif1α and anti-tubulin as indicated. Lower panel shows the densitometric ratio of Hif1α and tubulin.

To test Literature Lab on a more challenging, *in vivo *derived dataset, we applied Literature Lab to a set of genes whose expression increased with the prostate-specific expression of myristoylated AKT (also associated with the eventual prostate phenotype of prostatic intraepithelial neoplasm) and decreased when mice were treated with RAD001, an mTOR inhibitor (Fig. 2A). This experiment was well controlled, characterized in a published manuscript [17], and has publicly available data (Gene Expression Omnibus accession number GSE1413). We performed a 10-year literature query to identify pathway terms associated with the 64 genes previously found to have the expression pattern described above [17]. Literature Lab identified "Hif" and "Hypoxia" terms in association with the list of genes thus highlighting the key biological insight discussed in the primary report (Fig. 2B). While HIF gene expression (HIF1A and HIF3A) was part of the original gene list and contributed to the strong association, Literature Lab found 12 and 17 additional genes associated with the key terms "Hif" and "Hypoxia", respectively, thus elevating these terms above others. Subsequent Western blotting for protein expression of Hif1α further supports the identified association (Fig. 2C) and demonstrates Literature Labs potential utility for data derived from animal models.

### "Matrix metalloproteinase 9" and "VEGF" are associated with Breast Cancer Prognosis

Primary tumors perhaps provide the biggest challenge for analysis because of the additional associated technical and biological variation. We applied Literature Lab to the set of 70 genes associated with outcome in two seminal papers predicting breast cancer recurrence using microarray data [31,32]. Interestingly, of the cellular pathways investigated, "matrix metalloproteinase" and "VEGF" were strongly associated with the gene set (see Additional File 7). This unbiased association is strongly supported by prior studies finding MMP [35,36] and VEGF [37] activity strongly associated with the recurrent phenotype [37] and supports Literature Lab's applicability to data from human samples.

### Literature drift

Given the daily growth of biomedical literature, reproducible literature search results require investigators to designate a specific chronological interval within which they queried for associations between a gene list and key terms. However, repeated queries using identical search criteria within a fixed interval demonstrated some lability hereto forward referred to as "literature drift". In sequential runs over 5 months, we measured this drift using the absolute value of the percentage change in the LPF value. Literature drift resulted in a median difference between queries of 2.51%. This degree and rate of literature drift was dependent both on the gene list and key terms as three independent sets of genes queried for three sets of key terms experienced different degrees of drift (Table 1).

**Table 1:** Median of Absolute Value of Percentage Change in LPF over 5 Months

	***Metabolism Terms***	**Physiology Terms**	***Pathways Terms***
MPAKT Gene Set	1.90%	1.98%	3.09%
HL60 Gene Set	1.32%	3.08%	3.41%
vant Veer Gene Set	1.05%	2.22%	1.91%

By identifying the abstracts lost or gained between sequential runs, we identified a number of causal factors for literature drift:

• Changes to the various components of the NCBI search engine that result in different results for the same query. In particular, changes to the PubMed "phrase dictionary" are frequent and can yield different results for the same query at different points in time.

• The assignment of MeSH terms to abstracts subsequent to the addition of abstracts to the PubMed database.

• The editing of PubMed abstracts so as to change the title or text.

• The occasional deletion of abstracts from the database. Many of these deletions appear to be the removal of duplicates added to the PubMed database in error.

**Figure 3:**
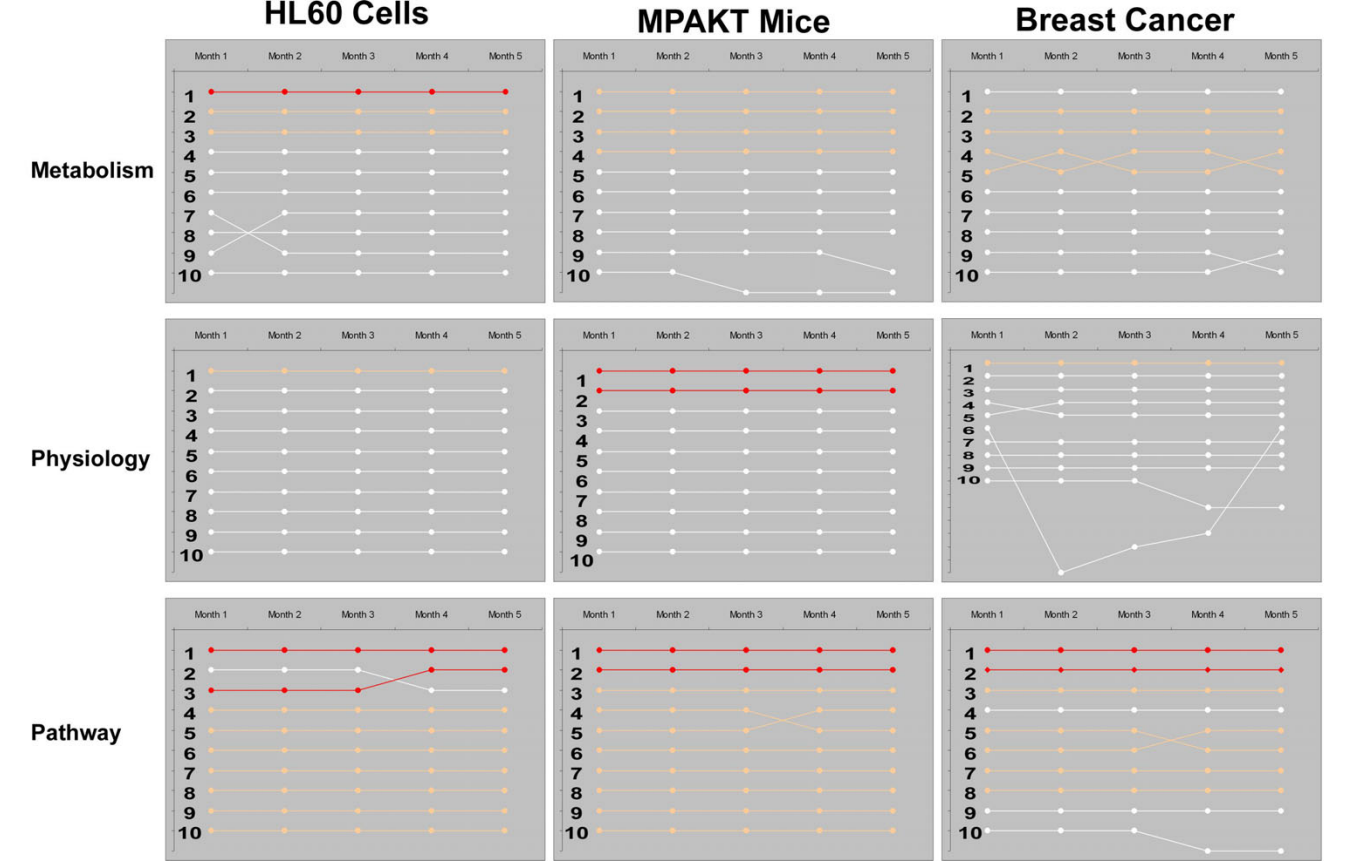
**Literature Drift**. Literature Lab results for repeat analysis performed on data from HL60 cells, MPAKT mice, and Breast Cancer tumors. The ranking of the top 10 terms from the Metabolism, Physiology, and Pathway lists are presented when the same analysis was performed within fixed dates (12/31/93 and 12/31/03). The ranking of each term is followed across the 5 monthly repeats by connecting lines. Color of line indicates strength of association (red = "strong"; tan = "moderate", white = no call).

Importantly, while literature drift affected the numbers of abstracts linking a gene list with a term list, it had only minor effects on the final results of Literature Lab (Fig. 3). In addition, when the effects of literature drift were analyzed according to the strength of association between a key term and a set of genes; stronger associations were less likely to change when compared to associations with modest or no weight (11% for Strong (1 of 9), 16% for Moderate (5/32), and 27% for associations without weight (13 of 49). Thus while literature drift impacts the number of associations identified between the medial literature and a set of genes, our methodology minimizes its impact.

### Discovery of increased FOSB in Metastatic Prostate Cancer

**Figure 4:**
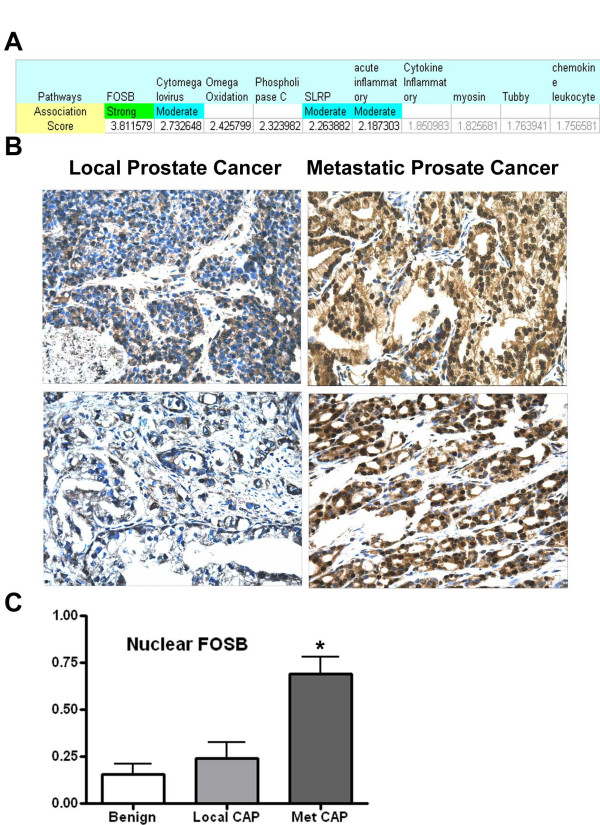
**FOSB Identification and Immunohistochemistry**. A) Ranking, association scores, and confidence calls for pathway terms associated with the 100 genes with increased expression in metastatic prostate tumors compared to local prostate tumors (Association score is the log of the product of frequency (logPF)). B) Two examples of local (left) and metastatic (right) prostate cancer FOSB expression measured by immunohistochemistry. C) Percentage of epithelial cell nuclei staining positive for benign prostate epithelium (n = 14), local prostate cancer (n = 20), and metastatic prostate cancer (n = 22) (* Met v. Local, p = 0.0013).

While the Literature Lab results from the HL60 cell line, MPAKT mouse, and breast cancer tumors are compelling, these sets had known biological associations and were used to help evaluate the methods herein described; they cannot be viewed as independent tests of our method. In order to test Literature Lab's ability to identify valid gene-key term associations through automated literature interrogation, we applied literature lab to microarray data obtained after RNA amplification of local and metastatic prostate cancer specimens. We first identified the top 100 genes up-regulated in the metastatic samples in order to identify the top pathway(s) associated with metastasis. FOSB was the top pathway and the only pathway term meeting heuristic criteria for a "strong" association (Fig. 4A). Expression of the FOSB gene was associated with metastasis but the additional identification of associations between FOSB and 5 other genes having increased expression in the metastatic tumors resulted in the top ranking of FOSB and a "strong" heuristic label. Based on this result, we performed FOSB immunohistochemistry on a tissue microarray containing benign, local malignant, and metastatic malignant prostate tissue. Nuclear FOSB was significantly increased in the metastatic tumors compared to the locally invasive tumors (p = 0.0013, two-tailed Mann Whitney test) thus confirming the association highlighted by Literature Lab (Fig. 4B and Fig 4C).

### Comparison with GOMiner, MESHER and GeneCite

To determine how Literature Lab compares with existing, publicly available sample annotation and literature mining technologies, we imported the AKT mouse and metastatic prostate cancer gene lists into GOMiner [21], MeSHer [20], and GeneCite [38]. When the AKT mouse gene set was imported into GOMiner, 222 GO terms were found to be significantly associated (p ≤ 0.05) with the genes differentially expressed (see Additional File 8). Among the significant GO terms were "response to hypoxia" and "glycolysis" thus demonstrating that GOMiner is able to identify similar underlying biology but these terms were among a large number of significant terms and not prominent. Here we note that GOMiner and Literature Lab represent different approaches (enrichment analysis v. Literature mining, respectively) and, as such, the results are not directly comparable. However, the purposes of Literature Lab and GOMiner are similar (i.e. the identification of biological processes implicated by differential gene expression) and our results suggest that direct literature mining and our statistical approach provide insight that cannot be fully reproduced using GOMiner.

MESHER did not find a significant association between "Hypoxia", "HIF", and "Hypoxia p53" and the AKT mouse gene list and there was very poor comparison between MESHER and Literature Lab despite each method using MeSH terms (see Additional File 9). GeneCite identifies the abstracts associating the terms and genes but has no direct measure of significance other than the number of abstracts (which favours more general terms over more specific terms). In addition, GeneCite has lower precision and recall when compared to Literature Lab due to the lack of a thesaurus for gene nomenclature (see Additional File 10). For example, many of the abstracts for the CAT gene refer to felines and CAT scans and not to the CAT gene.

When the genes differentially expressed between local and metastatic prostate cancers were imported into GOMiner and MESHER, there was poor overlap with both. FOSB, the term significantly associated with metastatic prostate cancer and subsequently validated by immunohistochemistry, was either not present in the library (GOMiner) or not associated with the gene list (MESHER, "Biogenetics-MeSH – "Genes, fos"). Results for GeneCite (see Additional File 11) exhibit the same limitations previously described for the AKT mouse gene set.

## Discussion

Full utilization of publicly available, data-rich resources remains a universal challenge in contemporary scientific investigation. As technologies have diminished the cost and time associated with data collection, content within diverse repositories of data have increased exponentially. The medical literature is one such data repository and a repository that continues to grow rapidly. While investigators frequently use computational tools to interrogate genomic or gene expression data repositories, few use similar tools when reviewing the literature.

Literature Lab represents a method to comprehensively interrogate the literature for associations between a list of genes and a list of key terms in an unbiased manner in order to highlight potentially important biological processes implicated by the gene list. While there are many methods by which to develop a gene list, we have designed Literature Lab to aid in the analysis of microarray experiments which frequently associate the expression of hundreds to thousands of seemingly unrelated genes with cellular behaviors, in vivo phenotypes, or disease outcomes. We developed and refined our methodology using gene sets from previously published work and successfully tested Literature Lab on a novel dataset. The pathway term FOSB was ranked highest by Literature Lab and highlighted as having a "strong" association; an increase in nuclear FOSB staining was subsequently confirmed with immunohistochemistry.

Literature Lab is complementary to the increasingly prevalent pathway oriented approaches to the analysis of microarray data (Reviewed in [39]). As a general approach, these methods look for significant differential expression within a microarray experiment using pre-determined aggregations of genes (alternatively called gene sets, metagenes, or gene modules) rather than individual genes [40]. Successful gene sets can identify underlying genetic abnormalities or signal transduction networks driving disease pathologies and help effectively bridge microarray data with biological significance [41,42].

Some pathway approaches methods use the literature and publicly available annotations (Gene Ontology) to develop gene sets and use these gene sets to interrogate expression data [43,44]. Literature Lab offers the opportunity to use a gene set derived from microarray data to interrogate the biomedical literature without a priori classification or annotation. As such, Literature Lab can appropriately interrogate the literature as it grows and evolves. When compared to two publicly available methods of analysis (GOMiner and MeSHer), the results of Literature Lab were more comparable with GOMiner. However, the statistical evaluation of associations identified by Literature Lab help improve the specificity of findings (highlighting strong associations) while maintaining sensitivity (neither GOMiner nor MeSHer identified the association between FOSB and genes with differential expression between local and metastatic prostate cancer). It should be noted, however, that given the difference in the approaches, our results cannot be interpreted as demonstrating the superiority of Literature Lab over GOMiner

Literature Lab remains dependent on the strength of the term lists and while we have demonstrated the use of lists for metabolism, physiology, and pathways, further development is focused on creating lists to include disease, pharmacological agents, drug toxicities, and many additional classes.

We initially anticipated that fixing the chronological interval for a query would ensure exact reproduction of the results. However, we identified literature drift within fixed retrospective intervals. While the degree of literature drift seems to range from minimal to moderate depending on the specific gene list, Literature Lab successfully limits the effects of literature drift especially for associations identified as "strong" with the current heuristics. Thus, while literature drift is unlikely to have significant impact on the associations identified by Literature Lab, some variation in the specific weights and rankings of associations will change even when investigators define a fixed chronological interval within which they perform their query.

For this initial description, we focused on developing a robust measure of association, a relatively useful measure of significance, and heuristic rules to highlight the most important associations. Clearly, the specifics of our methods will be the subject of further investigation and refinement. While we have identified some critical elements of success (avoiding measures of association that are driven by single gene-term associations and having a gene set size of 25 or more), work is ongoing to explore the effects of refining the genes based upon the statistical association between their expression and the phenotype, limiting Literature Lab to specific journals of high quality content, and increasing the number of sets of key terms with which to test the association between gene expression.

## Conclusion

The methodology herein described for Literature Lab highlights the biomedical literature as a content-rich resource amenable to automated, comprehensive interrogation. As with most complex data, successful comprehensive interrogation requires filtering out the noise and finding valuable information. Our current methods of gene annotation, key term curation, and literature interrogation, can find strong associations and are likely to benefit a diverse scientific community.

## Availability and Requirement

The instructions, software, and data required to perform an analysis of a gene list using the techniques described herein can be obtained from . Sun's Java Runtime environment Version 1.4 or higher is required in order to run the software (and may be downloaded from [22]). The software runs on both Windows platforms (Windows 2000 and later) and Linux platforms. Memory of 1 GB and 5 GB of available disk space are required (much of the disk space requirement is for temporary storage during the analysis). In addition, the results of the analysis are presented in a Microsoft Excel spreadsheet for viewing on any system having a spreadsheet viewer capable of rendering the Microsoft Excel format.

• **Project name**: Literature Lab

• **Project home page**: 

• **Operating system(s)**: Windows (2000 +) or Linux

• **Programming language**: e.g. Java

• **Other requirements**: Java Runtime environment 1.4 or higher

• **License**: Not required for Academic users

• **Any restrictions to use by non-academics**: License needed from Acumenta

## Authors' contributions

PGF oversaw all aspects of the research including development of the concept of Literature Lab, provision of microarray results for analysis by Literature Lab, evaluation of all results from Literature Lab, and drafting of the manuscript. MGM helped develop and implement the software for Literature Lab. DAS helped develop and implement Literature Lab. KS oversaw all experiments with the leukemia cells, provided microarray data, provided the NBT data, and helped in the interpretation of Literature Lab results. DDV performed, analyzed, and interpreted the FOSB immunohistochemistry on prostate cancer specimens. PRM participated in the development, conceptualization, and implementation of Literature Lab. ML oversaw the FOSB immunohistochemistry and helped with the interpretation of Literature Lab results. SCT lead the development of Literature Lab from conceptualization to implementation, performed all Literature Lab analyses, and helped draft the manuscript.

## Supplementary Material

Additional file 1 Supplemental Methods. Click here for file

Additional file 2 **Term Lists**. Excel spreadsheet of term lists used for physiology, metabolism, and pathway domains. Click here for file

Additional file 3 **Gene Thesaurus**. Excel spreadsheet of all genes with all aliases used for all experiments in this manuscript. Click here for file

Additional file 4 **Excluded Terms**. Excel spreadsheet of terms excluded from search due to ambiguity or limited associated information. Click here for file

Additional file 5 **Effects of Time Frame**. Standard Literature Lab interrogations performed on the HL60 gene set for key term lists for metabolism, pathways, and physiology. The only difference between the two interrogations involved time frame: One search included citations from the first 5 years of the standard 10 year interval (12/31/93 and 12/30/98, "1^st^5 yr") and the other the latter 5 years (12/31/98–12/31/03, "2^nd^5 yr"). Log_10_(PF) ("Score") was used to order key terms and standard heuristics were applied to identify associations as "strong" or "moderate". Click here for file

Additional file 6 **Effects of Gene Set Size**. Standard Literature Lab interrogations performed on the HL60 gene set for key term lists for metabolism with increasing size of the gene set. "Metabolism-X" where X equals 10, 25, 50, 100, 150, 200, 250, and 300 and represents the number of genes in the gene set.. Log_10_(PF) ("Score") was used to order key terms and standard heuristics were applied to identify associations as "strong" or "moderate". Click here for file

Additional file 7 **Results of Breast Cancer Gene Set**. The 70-genes associated with outcome for localized breast cancer were used as a gene set and interrogated by Literature Lab for associations between members of the gene set and key terms included on the lists of Physiology, Metabolizm, and Pathways. Log_10_(PF) ("Score") was used to order key terms and standard heuristics were applied to identify associations as "strong" or "moderate". Click here for file

Additional file 8 **GOMiner**. Excel spreadsheet of results from GOMiner using gene list from MPAKT experiment annotated for overlap with Literature Lab. Click here for file

Additional file 9 **Mesher**. Excel spreadsheet of results from MESHer using gene list from MPAKT experiment annotated for overlap with Literature Lab. Click here for file

Additional file 10 **GeneCite MPAKT**. Excel spreadsheet of results from GeneCite using gene list from MPAKT experiment annotated for overlap with Literature Lab. Click here for file

Additional file 11 **GeneCite Metastatic Prostate Cancer**. Excel spreadsheet of results from GeneCite using gene list from local v metastatic prostate cancer experiment annotated for overlap with Literature Lab. Click here for file
