# Supplementary material for: Literature Lab: a method of automated literature interrogation to infer biology from microarray analysis
Source: BMC Genomics. 2007 Dec 18;8:461. doi: 10.1186/1471-2164-8-461 (PMC2244637; doi:10.1186/1471-2164-8-461)
Supplement: Additional file 1 — Supplemental Methods. [file 1471-2164-8-461-S1.doc]

# Supplemental Methods

**The application of automated literature interrogation to infer cancer biology from microarray analysis**.

Phillip G. Febbo^1^, Mike G. Mulligan^2^, David A. Slonina^2^, Pradip Majumder^3^, Kimberly Stegmeir^4^, Dolores Di Vizio^5^, Mark A. Rubin^5^, Paul R. Martinez^2^, Massimo Loda^5^, and Stephen C. Taylor^2^

## Gene Annotation

A component of a commercial software product, the Acumenta Gene Thesaurus® was used to obtain appropriate terms to locate text mentioning a specific gene. The Acumenta Gene Thesaurus® used for the analyses consisted of all of the genes having UniGene Cluster IDs as listed on Stanford University’s SOURCE web site (<http://genome-www5.stanford.edu/cgi-bin/source/sourceSearch>). The Thesaurus consists of the list of terms (gene symbols, gene names, and “aliases”) for each gene drawn from the Stanford Source database. The terms for each gene are tested by performing searches of PubMed using an OR of the terms. When the Thesaurus was originally developed, these tests determined that:

- Terms consisting entirely of numerals or numerals and punctuation resulted in very poor precision because of the way in which the PubMed search engine handles such queries. Likewise terms of the form “EC.numeral.numeral….” (a protein identifier) performed poorly and were ignored.
- Terms of three characters or less likewise generally resulted in very poor precision. The use of short acronyms in scientific articles is so common that most terms of three characters or less have multiple meanings. Three character or less terms were not used to locate abstracts for a gene unless either:
  - The three character or less term was on a list of terms known to have good precision
  - The three character or less term was the only synonym for the gene that found abstracts.
- Terms that happen to be included in the NLM MeSH Thesaurus frequently result in very poor precision with respect to identifying articles specifically about a gene.

The Thesaurus is curated by running tests of the gene terms excluding the aforementioned numeric terms and short terms. These tests search for the terms as “Text Words” on PubMed in order to avoid the complication of having the term inappropriately be interpreted as a MeSH term. Short terms of three characters or less are used in the event that no other terms for a gene identify any abstracts for the gene. The results of these tests are manually reviewed to exclude specific gene terms that result in a precision of less than 70% and to include specific three character terms that do not damage the precision of the results to below 70%. Given the extremely voluminous results, not all genes are manually reviewed. All genes whose terms identified more than 500 abstracts on PubMed plus a list of g-Protein Coupled Receptor genes and a list of Kinase related genes are reviewed. The most recent 20 to 50 abstracts are examined to determine if the 70% precision is being achieved. If not, the specific terms most significantly damaging the precision are removed until 70% precision is observed. If a gene has a three character or less term that does not damage precision below 70%, it is re-added to the Thesaurus.

The Acumenta Gene Thesaurus® is updated frequently as changes are made to the Stanford SOURCE database and now incorporates nomenclature from other databases such as Entrez Gene,OMIM, HGNC, and UniProt. However, for purposes of the experiments described herein, a constant version of the Gene Thesaurus as of 12/30/2003 was used so that results of experiments run at different times would be comparable. The gene thesaurus and excluded terms are provided in Supplemental Tables II and III, respectively.

## Topic Set Curation

Most of the topic sets used in the experiments were defined using terms from the MeSH Thesaurus provided by the National Library of Medicine. The list of terms for each topic set consisted of all of the appropriate descendents of the selected topic set as listed in MeSH. The topic sets used in the experiments were Cell Lines (Cells, Cultured) [A11.251], Cell Structures [A11.284], Cell Types[A11] (excluding Cells, Cultured [A11.251] and Cell Structures [A11.284]), Diseases[C], Metabolism [G06.535], and Cell Physiology [G04.335]. The codes in brackets are the MeSH IDs for the parent each topic set. A few of the descendents of these MeSH terms were ignored because very few abstracts were associated with the term. A complete list of the MeSH terms used can be found in Supplemental Table I.

When using MeSH terms to search PubMed, the search syntax used “MeSH Subheading Explosion” so that the resulting list of abstracts included abstracts coded with descendents (if any) of the MeSH term as well as abstracts coded with the MeSH term itself. Some time after abstracts are added by the NLM to the PubMed database, each abstract is associated with the relevant MeSH terms as “metadata” in order to assist in the location of abstracts associated with a particular topic or concept. MeSH is an ontology containing hierarchical relationships. For example, the MeSH term Phosphorylation [G06.535.790] has as children Oxidative Phosphorylation [G06.535.790.550] and Photophosphorylation [G06.535.790.605]. Abstracts relating to phosphorylation in general are associated with Phosphorylation [G06.535.790]. Abstracts relating to oxidative phophorylation are associated with Oxidative Phosphorylation [G06.535.790.550] but, generally, not associated with the parent term. Our analysis of the abstracts associated with a specific term counted and identified the abstracts associated directly with the term as well as those associated with any descendent of the term but did not multiply count abstracts associated with a parent and with one or more descendents. Thus, in order to identify all of the abstracts associated with phosphorylation, we included all of the abstracts associated with Oxidative Phosphorylation [G06.535.790.550] and Photophosphorylation [G06.535.790.605] as well as those associated directly with Phosphorylation [G06.535.790]. The use of MeSH explosion in this way is consistent with the methodology used by the NLM to associated terms in MeSH with abstracts. Furthermore, because the measure of the strength of association between a single term and a list of genes (PF) is independent of the association of any other term with the list of genes, our analysis is not adversely effected by the inclusion of terms at different conceptual levels in the topic list.

An additional topic set, Pathways, was developed through an examination of the pathway descriptions from BioCarta ([http://www.biocarta.com/genes/allPathways.asp](http://www.biosys.com/)). A long list of general terms commonly used in pathway descriptions was first generated through an examination of these BioCarta pathway records and from an examination of numerous PubMed abstracts using the term “pathway”. A list of pathways and pathway specific terms was determined, again through an examination of the BioCarta pathway records supplemented by other pathways mentioned in PubMed abstracts using the term “pathway”. The resulting term lists were then tested by performing searches on PubMed as searches for (OR of pathway specific terms) AND (OR of generic pathway terms). These results were reviewed and the terms adjusted with a target precision of 70% in the search results. A complete list of the Pathway topic set terms can be found in Supplementary Table I.

## Literature Search

A list of the PubMed abstract ID’s for each gene and each topic were obtained by searching PubMed using the appropriate terms and a constant date range as described below. The PubMed Electronic Date (EDAT) was used to query a constant subset of the PubMed abstracts.

### Gene Search

A search for a gene consisted of the following query using the valid terms in the Acumenta Gene Thesaurus®. With T_1_ through T_N_ representing the valid terms for a gene, D_1_ representing the start date, and D_2_ representing the end date, the following query was used to obtain the PubMed abstract IDs for a gene:

(T_1_[TW] OR T_2_[TW] OR … T_N_[TW]) AND “D_1_”[EDAT] : “D_2_”[EDAT]

When a gene term contained punctuation characters that cause problems in the PubMed search engine, the term was placed within quotation marks in the query.

### Topic Search (MeSH)

A search for a topic consisted of the following query using the MeSH term(T_1_). With D_1_ representing the start date, and D_2_ representing the end date, the following query was used to obtain the PubMed abstract IDs for a topic:

T_1_ AND “D_1_”[EDAT] : “D_2_”[EDAT]

### Topic Search (Pathway Topic)

A search for a pathway topic consisted of the following query using the MeSH. With T_1_ through T_N_ representing the valid terms for a topic, D_1_ representing the start date, and D_2_ representing the end date, the following query was used to obtain the PubMed abstract IDs for a topic:

(T_1_ OR T_2_ OR … T_N_) AND “D_1_”[EDAT] : “D_2_”[EDAT] AND (communication[TW] OR cleavage OR cascade OR cascades OR signal OR signals OR signaling OR signalling OR pathway OR pathways OR activation OR biosynthesis OR mechanism of action[TW] OR regulate OR regulation[TW] OR transduction OR trafficking). The final portion of this query represents the list of generic terms that are common to abstracts about pathways. Because certain of the terms, such as “communication”, are MeSH terms having non-pathway related meaning, the search uses them exclusively as PubMed “Text Words”.

## Supplemental Materials and Methods

The goal of the Acumenta Literature Lab is to suggest one or more topics from several biological domains (cell type, metabolism, disease, etc). We treat each domain independently. Within a domain, we want to calculate a numeric score that measures the strength of the association between a target gene set and each topic. The association we are measuring comes from counting PubMed abstracts that mention one or more of the genes in the target set and any of the domain topics.

It became apparent that some normalization was required for the citation frequency of the term used due to the large disparity between the number of total abstracts associated with any single term. Thus, we initially used a simple ratio of the number of unique abstracts containing any of the genes from the list and a given term divided by the total number of abstracts containing the given term. While this de-emphasized commonly used terms, it placed too large an emphasis on rare terms that could be ranked highly based on a single abstract. Thus, we decided to apply a previously published[1]method of measuring the association between genes and terms known as the product of frequencies (PF). The PF is the product of the proportion of term abstracts mentioning the gene and the proportion of the gene abstracts mentioning the term.

For a particular topic, let *T* be the number of PubMed abstracts that mention that topic, let *G* be the number of abstracts that mention any of the genes in our target gene set, and let *A* be the number of abstracts that mention both the topic and any target gene (the size of the set that is the Boolean AND of the first two sets of abstracts).

We have examined four different scoring metrics. The first two measures are based on the three counts: *T*, *G*, and *A*. The second two look at the first two scores relative to the mean and standard deviation of the distribution of topic scores from weighted random gene sets of the same number of genes.

### Expected abstracts

The first score looks at the number of gene set AND topic abstracts, relative to the number of topic abstracts:

$$EA=\frac{A}{T}$$

### Product of frequencies

The second score takes in to account the number of abstract mentions against the entire target gene set. Let *G_g_* be the number of abstracts for the single gene *g* from the gene set, and let *A_g_* be the number of abstracts for *g* and the topic. The following score credits *A_g_* being larger relative to either or both *G_g_* and *T*.

$$PF=\sum_{g\in geneset} \frac{A_{g}}{G_{g}}\times\frac{A_{g}}{T}$$

### Scores relative to standard deviation

Any score applied across the domain topics will create a ranking of those topics. We want to provide a sense for how significant the association is between the gene set and the top ranked topics. We do this by comparing each topic’s score against a distribution of scores between that topic and randomly selected gene sets. If the target gene set’s score is at the top of the random distribution, then we believe we have an interesting association.

$$EA_{\sigma}=\frac{\left(EA-mean\right)}{stddev}$$

When we construct random gene sets and produce a distribution of *PF* scores, we find that by taking the *log(PF)* we more closely approximate a normal distribution, justifying the use of the mean and standard deviation. The final LPF ranking score used by the literature lab is based on the *mean* and *standard deviation* statistics derived from this $\log PF$ distribution.

$$LPF_{\sigma}=\frac{\left(\log\left(PF\right)-mean\right)}{stddev}$$

The significance of the strength of the relationship between an experimental gene set and a term was measured by comparing the log (base 10) of the sum of the PF values for the experimental gene set (called the LPF) with a distribution of the same values for 1000 random gene sets of the same size as the experimental gene set. A score representing the number of standard deviations above or below the mean value for the random gene sets was assigned to each term and the terms then ranked in decreasing order of this score.

A review of the ranked terms for the various topic lists and gene sets demonstrated that the high rank did not always indicate a particularly strong relationship between the term and the set of genes as a set. This could occur when:

- The high score was due exclusively to a relationship between a term and one or a very small number of genes in the gene set.
- The distribution of scores (PF) for the random gene sets was severely skewed for a particular term.
- There was very little evidence (as measured by the total number of abstracts involved) for the relationship.

As a result, we developed some heuristic rules for labeling the relationships as “Strong” or “Moderate” based upon the data in the MPAKT gene set where hypoxia and some other terms were known to have a strong relationship with the gene set. This work resulted in the following definitions:

1) A “Strong” relationship requires:

1. LPF score for the gene set to be at least 3.0 standard deviations above the mean LPF for the 1000 random gene sets.
2. LPF score for the gene set had to rank in the top 25 of the 1000 random gene sets.
3. At least 3 genes in the gene set must have a non-zero LPF.
4. There must be at least 15 unique abstracts underlying the LPF for the gene set.

2) A “Moderate” relationship requires:

1. LPF score to be at least 2.0 standard deviations above the mean LPF for the 1000 random gene sets.
2. LPF score for the gene set had to rank in the top 50 of the 1000 random gene sets.
3. At least 2 genes in the gene set must have a non-zero LPF.
4. There must be at least 10 unique abstracts underlying the LPF for the gene set.

The seemingly redundant (and more liberal when compared to the first rule) score ranking requirement is designed to handle cases where there are very few abstracts associated with a term regardless of any gene mentions. In these situations, a significant number of the random gene sets can be null sets and the subsequent score distributions are erratic.

## Effects of Time Interval and Gene Number

During the development of Literature Lab we performed additional analyses to explore the sensitivity of our literature analysis techniques multiple parameters (see Supplemental Methods). For the HL60 results, we specifically assessed how the time frame of query and the size of the interrogated gene sets effects results. When the scores for metabolism, physiology, and pathway topic lists are computed using the first five years and last five years of the ten-year period initially interrogated similar associations between gene sets and key terms are found albeit with modest changes order and confidence scores (Supp. Fig. 1). In contrast, a series of computations using gene sets of increasing sizes (e.g. using the n most differentially expressed genes, where n = 10, 25, 50, 100, 150, 200, 250 and 300), demonstrates that the size of the gene set can have significant effects on the results (Supp Fig. 2). The term associations and term rankings stabilized at a gene set size of 150 with gene sets having fewer members demonstrating greater variability. Both the time interval and gene number tests were applied to two additional experimentally derived gene sets with similar results. While these tests demonstrate that using larger gene sets yields better results, the analyses on the smaller gene sets do identify most of the significant terms.

## Literature Drift

Given the daily growth of biomedical literature, reproducible literature search results require investigators to designate a specific chronological interval within which they queried for associations between a gene list and key terms. However, repeated queries using identical search criteria within a fixed interval demonstrated some lability hereto forward referred to as “literature drift” (see Fig. 1A for an example). In sequential runs over 5 months, we measured this drift using the absolute value of the percentage change in the LPF value. Literature drift resulted in a median difference between queries of 2.51%. This degree and rate of literature drift appears dependent on the gene list or key terms as three independent sets of genes queried for three sets of key terms experienced different degrees of drift (Table 1).

#### Table 1. Median of Absolute Value of Percentage Change in LPF over 5 Months

|  | Metabolism Terms | Physiology Terms | Pathways Terms |
| --- | --- | --- | --- |
| MPAKT Gene Set | 1.90% | 1.98% | 3.09% |
| HL60 Gene Set | 1.32% | 3.08% | 3.41% |
| vant Veer Gene Set | 1.05% | 2.22% | 1.91% |

By identifying the abstracts lost or gained between sequential runs, we identified a number of causal factors for literature drift:

- Changes to the various components of the NCBI search engine that result in different results for the same query. In particular, changes to the PubMed “phrase dictionary” are frequent and can yield different results for the same query at different points in time.
- The assignment of MeSH terms to abstracts subsequent to the addition of abstracts to the PubMed database.
- The editing of PubMed abstracts so as to change the title or text.
- The occasional deletion of abstracts from the database. Many of these deletions appear to be the removal of duplicates added to the PubMed database in error.

Importantly, while literature drift affected the numbers of abstracts linking a gene list with a term list, it had only minor effects on the final results of Literature Lab (Fig 1B). In addition, when the effects of literature drift were analyzed according to the strength of association between a key term and a set of genes; stronger associations were less likely to change when compared to associations with modest or no weight (11% for Strong (1 of 9), 16% for Moderate (5/32), and 27% for associations without weight (13 of 49). Thus while significant literature drift occurs, our methodology is sufficiently robust so as to minimize its effects and associations identified as being strong using the current heuristics are less sensitive to literature drift.

***Immunohistochemistry***

A prostate tissue microarray containing samples of benign prostate epithelium (n = 14), locally invasive prostate adenocarcinoma (n = 20), and metastatic prostate adenocarcinoma (n = 22) were stained for FOSB. The FOSB staining was performed as previously described [2, 3]. Briefly, 5 micrometer sections were cut from the TMA block, deparaffinized, rehydrated and subjected to microwaving in 10-mM Citrate buffer (pH 6.00) in a 750W owen, for 15 minutes. The polyclonal anti-FOSB primary antibody (Santa Cruz Biotechnologies, Inc.), was incubated (1:50 dilution) at room temperature in an automated stainer (Optimax Plus 2. 0; Biogenex, San Ramon, CA). Antigen-antibody reaction was revealed with standardized development times, using the Streptavidin method with 3, 3 diaminobenzidine as substrate. Meyer Hematoxylin was used as nuclear counterstaining. FOSB nuclear positivity was scored on a total of 50 nuclei per sample. The statistical difference in FOSB staining between the unpaired populations was assessed using a two-tailed Mann Whitney test (GraphPad Prism4 Software).

***GeneCite Testing***

The GeneCite software as downloaded from the Walter Reed Army Institute of Research web site (<http://wrair-www.army.mil/index.php?view=tools>) experienced fatal errors when first run with the lists of gene symbols and terms. To identify the errors, the software modules (the function of which was to write the final output data to disk) exhibiting the failures were de-compiled and examined. The cause of the errors was a variable not initialized when PubMed did not respond to queries in an expected fashion. The failing software modules were modified so as to not fail in these circumstances but to proceed with the writing of the results after initializing the appropriate variable with a value (-1) indicating that the specific gene/term pair query had failed. The missing data was subsequently recovered by using GeneCite for the single gene/term combinations that had failed.

1. Hu, Y., et al., *Analysis of genomic and proteomic data using advanced literature mining.* J Proteome Res, 2003. **2**(4): p. 405-12.

2. Loda, M., et al., *Expression of mitogen-activated protein kinase phosphatase-1 in the early phases of human epithelial carcinogenesis.* Am J Pathol, 1996. **149**(5): p. 1553-64.

3. Rossi, S., et al., *Fatty acid synthase expression defines distinct molecular signatures in prostate cancer.* Mol Cancer Res, 2003. **1**(10): p. 707-15.
