# Supplementary figures and images for: Literature Lab: a method of automated literature interrogation to infer biology from microarray analysis
Source: BMC Genomics. 2007 Dec 18;8:461. doi: 10.1186/1471-2164-8-461 (PMC2244637; doi:10.1186/1471-2164-8-461)

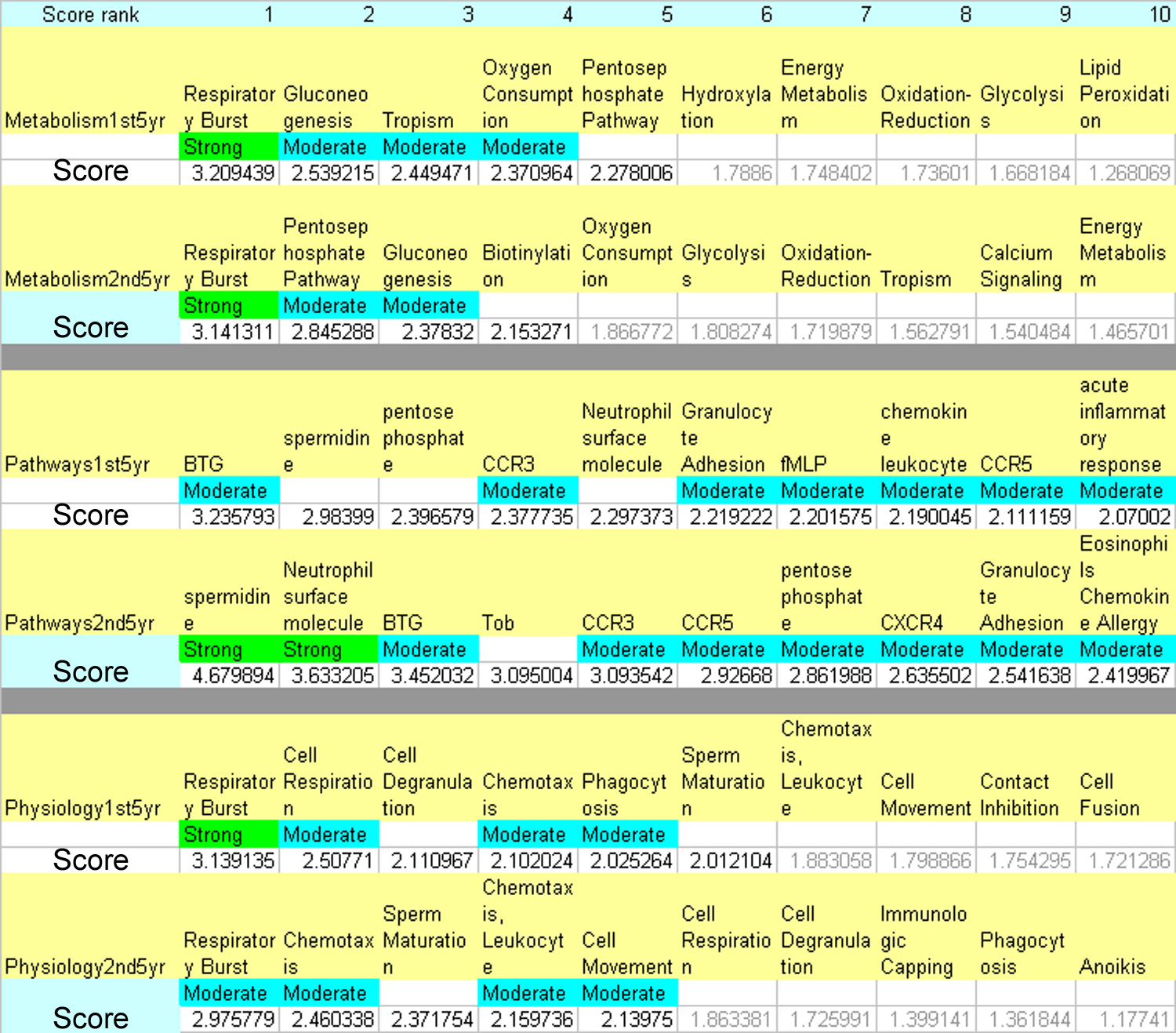

Supplement: Additional file 5 — Effects of Time Frame. Standard Literature Lab interrogations performed on the HL60 gene set for key term lists for metabolism, pathways, and physiology. The only difference between the two interrogations involved time frame: One search included citations from the first 5 years of the standard 10 year interval (12/31/93 and 12/30/98, "1st5 yr") and the other the latter 5 years (12/31/98–12/31/03, "2nd5 yr"). Log10(PF) ("Score") was used to order key terms and standard heuristics were applied to identify associations as "strong" or "moderate". [file 1471-2164-8-461-S5.JPEG]

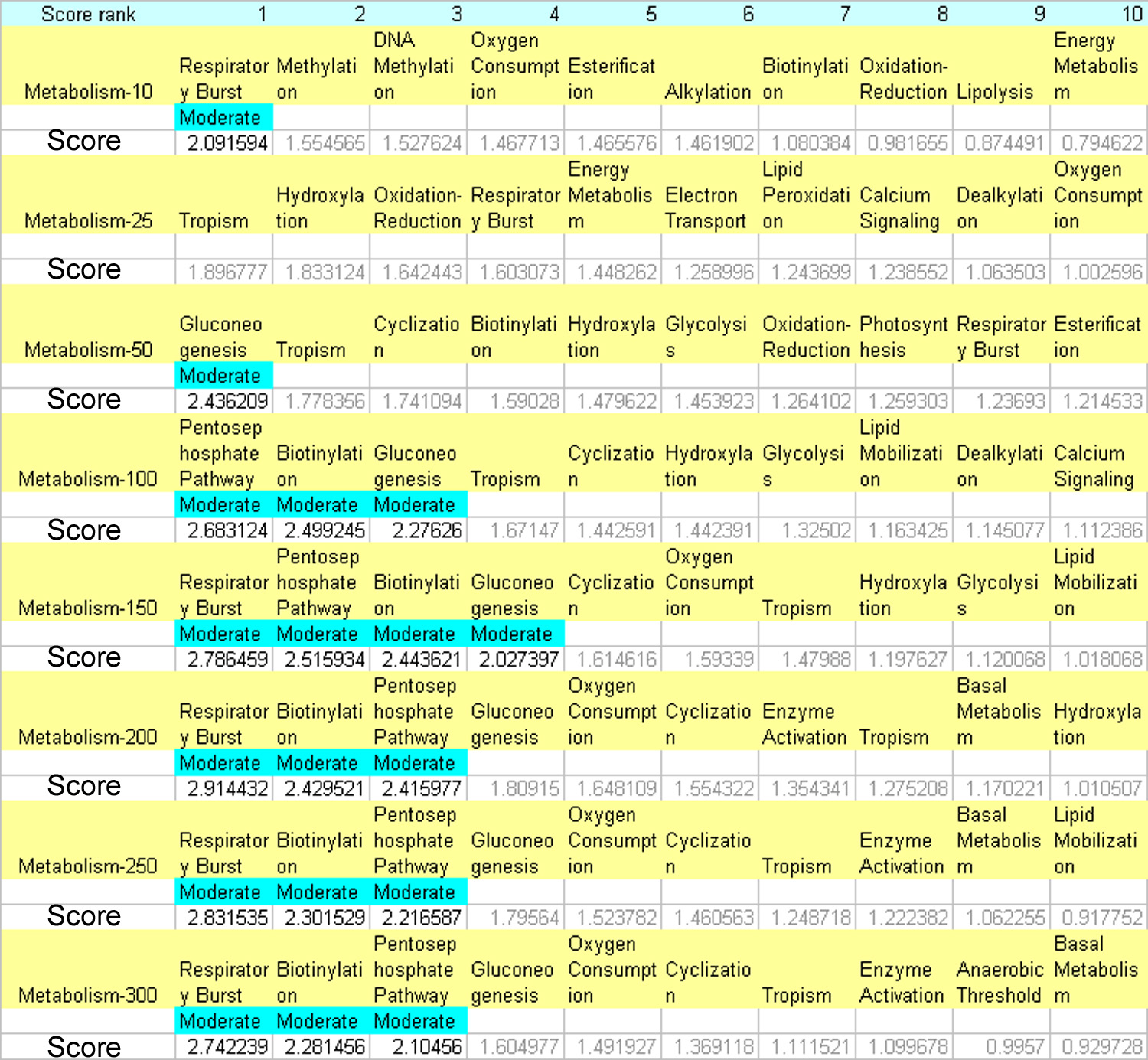

Supplement: Additional file 6 — Effects of Gene Set Size. Standard Literature Lab interrogations performed on the HL60 gene set for key term lists for metabolism with increasing size of the gene set. "Metabolism-X" where X equals 10, 25, 50, 100, 150, 200, 250, and 300 and represents the number of genes in the gene set.. Log10(PF) ("Score") was used to order key terms and standard heuristics were applied to identify associations as "strong" or "moderate". [file 1471-2164-8-461-S6.JPEG]

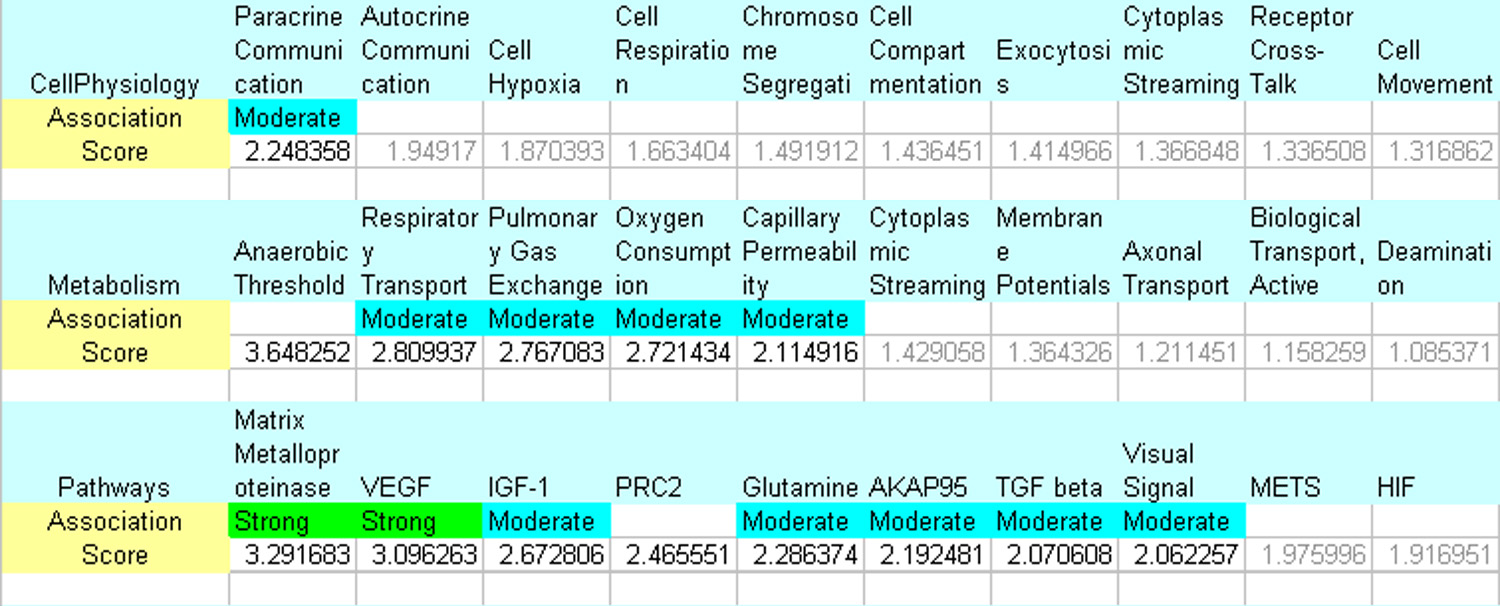

Supplement: Additional file 7 — Results of Breast Cancer Gene Set. The 70-genes associated with outcome for localized breast cancer were used as a gene set and interrogated by Literature Lab for associations between members of the gene set and key terms included on the lists of Physiology, Metabolizm, and Pathways. Log10(PF) ("Score") was used to order key terms and standard heuristics were applied to identify associations as "strong" or "moderate". [file 1471-2164-8-461-S7.JPEG]
